# Network analysis of grade II into grade III transition in rectum cancer patients 

**Published:** 2018

**Authors:** Mohammad Rostami-Nejad, Vahid Mansouri, Reza Mahmoud Robati, Hamid Mohaghegh Shalmani, Reza Mahmoudi Lamouki, Mostafa Rezaei Tavirani

**Affiliations:** 1 *Gastroenterology and Liver Diseases Research Center, Research Institute for Gastroenterology and Liver Diseases, Shahid Beheshti University of Medical Sciences, Tehran, Iran.*; 2 *Proteomics Research Center, Faculty of Paramedical Sciences, Shahid Beheshti University of Medical Sciences, Tehran, Iran.*; 3 *Skin Research Center, Shahid Beheshti University of Medical Sciences, Tehran, Iran.*; 4 *Basic and Molecular Epidemiology of Gastrointestinal Disorders Research Center, Research Institute for Gastroenterology and Liver Diseases, Shahid Beheshti University of Medical Sciences, Tehran, Iran. *

**Keywords:** Rectum, Cancer, Gene, Protein

## Abstract

**Aim:**

Finding important differential genes between grade II and grade III of rectum cancer was the aim of this study.

**Background:**

Colorectal (CRC) cancers (CRC) are known as the third diagnosed cancer and the second leading to death cancers. Life style is an important risk factor of CRCs. Diagnosis of rectum cancer estimated as 44% of colon cancer.

**Methods:**

Differentially expressed genes (DEGS) related to grade II into grade II in 6 patients are retrieved from gene expression omnibus (GEO) and investigated by protein-protein interaction (PPI) network analysis. Central nodes of the network are identified and enriched to determine biochemical pathways. Action map is illustrated for the central genes.

**Results:**

Among 15 central genes including AKT1, PRDM10, GAPDH, TP53, SRC, EGFR, ALB, INS, CTNNB1, EGF, IL6, RHOA, DECR1, ACACA, GMPS role of AKT1 is highlighted due to prominent role in the integrity of the network and participation in the most determined pathways. However, significant regulatory effect of INS, AKT1, EGF, EGFR, and CTNNB1 is tinted in action map.

**Conclusion:**

It seems that AKT1, EGFR, and TP3 are suitable drug targets to prevent rectum cancer progression.

## Introduction

 Colorectal cancers (CRC) are classified as the third diagnosed cancer and the second leading to death among cancers. Investigation indicates that special life style is an important risk factor of CRCs. High fruit and vegetable diet, maintenance of body weight, in oppose with Tobacco consuming, high red meat diet, type II diabetes mellitus, aging, and family history of CRC reduce the risk of CRCs. However, sex is counted as risk factor of CRC, an investigation attributed it to common pathway of colon cancer and breast cancer in women (1, 2). Numbers of 43030 individuals including 25920 male and 17110 female estimated new cases with rectum cancer were reported in US in 2018. In this report, the amount of rectum cancer in patients were estimated as 44% of colon cancer patients (3). Since treatment of cancer in advanced stages is difficult, early detection of disease or at least in the lower stages is a main effort in clinic (4). 

High through output methods like proteomics and genomics are used frequently to detect biomarkers related to early stages of different cancers (5, 6). In this case, large numbers of differentially expressed genes (DEGS) are highlighted and the dependent metabolites or proteins are analyzed (7). There is a useful method to screen the introduced differential agents to find the best and relevant ones to the disorder. This method is PPI network analysis which is able to interact the query genes (or protein and also metabolites) in a interacted unit as interactome (8). Central properties of interactome or network differentiate the interacted genes and it is possible to extract critical genes among the large numbers of query genes (9). Degree, betweenness centrality, and closeness centrality are three important central parameters of the nodes (10). In this approach, it is possible that regulatory effects of genes on each other be investigated. Activation, inhibition, and also expression regulation including up and down-regulations are three action features of the studied genes which can be identified in network analysis (11). In this study differential expressed genes that distinct grade II and grade III rectum cancer are evaluated by PPI network analysis to find possible drug targets to prevent grade II into grade III transition in the rectum cancer patients. 

## Methods

Gene Expression Omnibus database is a valuable source of gene expression data. Gene expression profile of 6 individual’s samples characterized by GSE25071/GPL2986 were acquired. Gene expression profiles of three grade II rectum cancer patients including two female (53 and 48 years old) and a 44 years old male and three grade III rectum cancer patients including two female (41 and 45 years old) and a 40 years old male have been chosen. The samples are presented as GSM615925, GSM615933, GSM615946, GSM615941, GSM615947, and GSM615929. The top 250 significant and characterized top DEGs were selected and interacted via PPI network analysis by Cytoscape software version 3.6.0 (12). P-value less than 0.05, and fold changes more than 2 and less than 0.5 were considered. The network was analyzed by Network analyzer an application of Cytoscape. The central nodes based on centrality parameters including degree (cut off mean+2SD), betweenness centrality (cut off %5 of top nodes), and closeness centrality (cut off %5 of top nodes) were determined (13). Action network including expression, activation, and inhibition relationships related to the central nodes was constructed via CluePedia. Biochemical pathways associated to the central nodes were introduced from KEGG by ClueGO. Distribution of central nodes among the pathways was analyzed. Interaction pattern of central nodes was prepared by Cytoscape. 

## Results

**Figure 1:**
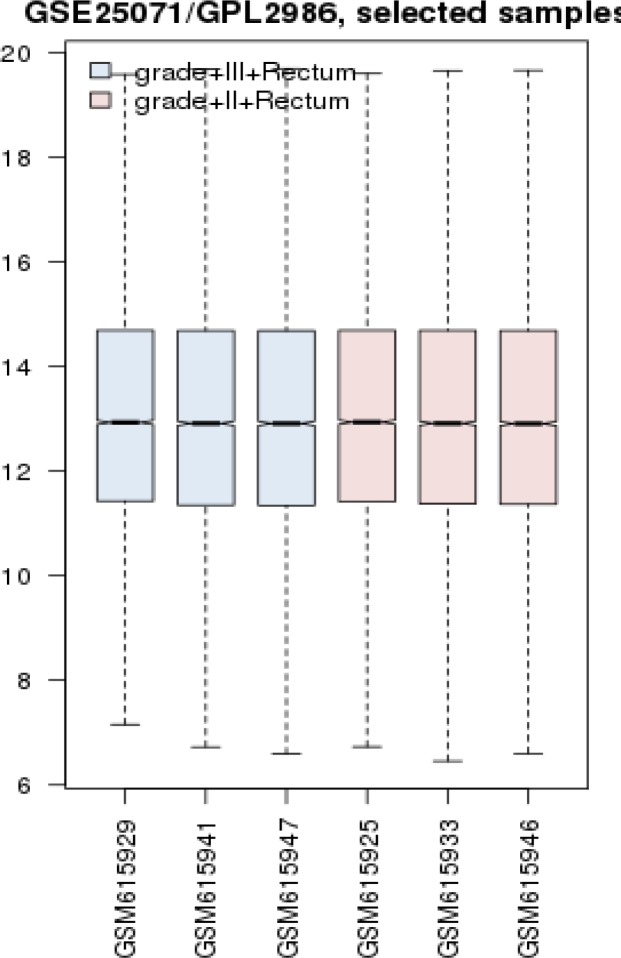
Box plot comparison of samples via GEO2R

The 6 samples were matched via box plot analysis by GEO2R (see figure 1). The gene expression profiles are median centered, therefore they are comparable. As it is depicted in the figure 1 the quarters show approximately symmetry. 

**Figure 2:**
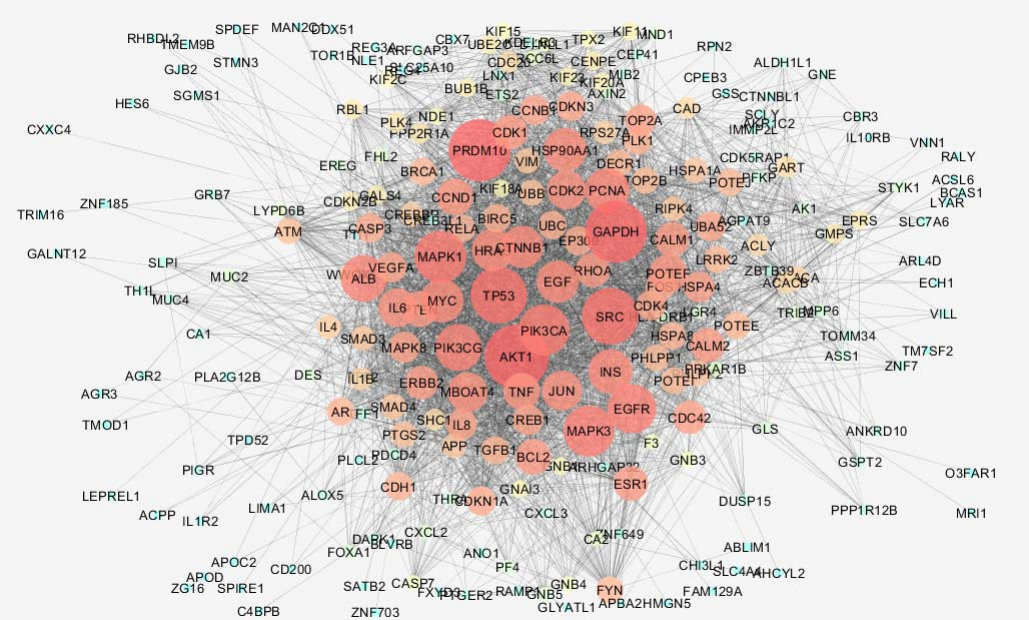
Main connected component of PPI network related to grade II - grade III transition of rectum cancer. The nodes are layout based on degree value

**Table 1:** Hubs (yellow colored), bottlenecks (green colored), and top nodes based on closeness value of human grade II – grade III transition of rectum disease

R	Disply name	discription	Degree	BC	CC
1	AKT1	v-akt murine thymoma viral oncogene homolog 1	104	0.04	0.62
2	PRDM10	PR domain containing 10	102	0.03	0.61
3	GAPDH	glyceraldehyde-3-phosphate dehydrogenase	102	0.02	0.61
4	TP53	tumor protein p53	97	0.02	0.60
5	SRC	v-src sarcoma (Schmidt-Ruppin A-2) viral oncogene homolog (avian)	97	0.02	0.60
6	EGFR	epidermal growth factor receptor	90	0.02	0.59
7	ALB	albumin	88	0.03	0.59
8	INS	insulin	86	0.04	0.59
9	CTNNB1	catenin (cadherin-associated protein), beta 1, 88kDa	84	0.03	0.58
10	EGF	epidermal growth factor	84	0.02	0.57
11	IL6	interleukin 6 (interferon, beta 2)	81	0.02	0.57
12	RHOA	ras homolog family member A	77	0.02	0.56
13	DECR1	2,4-dienoyl CoA reductase 1, mitochondrial	61	0.02	0.54
14	ACACA	acetyl-CoA carboxylase alpha	48	0.02	0.51
15	GMPS	guanine monphosphate synthetase	37	0.02	0.46

**Figure 3:**
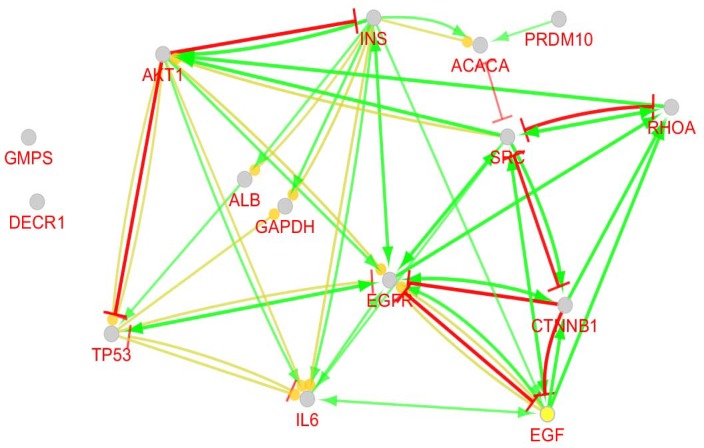
Expression action including up – regulation (yellow colored arrow with round tip) and down – regulation (yellow arrow with vertical bar tip), activation (green colored arrow) and inhibition (red colored arrow) actions related to 15 central nodes. CluePedia was applied to analyze actions

**Table 2:** Biochemical pathways (analyzed by ClueGO) related to the central nodes were found in KEGG_20.11-2017. %G/T and G/T refer to percentage of gene contribution in TEM and gene per term, respectively

R	GOTerm	GO Groups	% G/T	G/T	Associated Genes Found
1	HIF-1 signaling pathway	Group0	6.00	6	[AKT1, EGF, EGFR, GAPDH, IL6, INS]
2	Adherens junction	Group1	5.56	4	[CTNNB1, EGFR, RHOA, SRC]
3	Prolactin signaling pathway	Group2	4.29	3	[AKT1, INS, SRC]
4	Colorectal cancer	Group3	5.56	4	[AKT1, CTNNB1, RHOA, TP53]
5	ErbB signaling pathway	Group4	4.65	4	[AKT1, EGF, EGFR, SRC]
6	Pancreatic cancer	Group4	5.33	4	[AKT1, EGF, EGFR, TP53]
7	Endometrial cancer	Group4	8.62	5	[AKT1, CTNNB1, EGF, EGFR, TP53]
8	Glioma	Group4	5.63	4	[AKT1, EGF, EGFR, TP53]
9	Prostate cancer	Group4	6.19	6	[AKT1, CTNNB1, EGF, EGFR, INS, TP53]
10	Melanoma	Group4	5.26	4	[AKT1, EGF, EGFR, TP53]
11	Bladder cancer	Group4	9.76	4	[EGF, EGFR, SRC, TP53]
12	Non-small cell lung cancer	Group4	6.06	4	[AKT1, EGF, EGFR, TP53]
13	Central carbon metabolism in cancer	Group4	4.62	3	[AKT1, EGFR, TP53]

Numbers of 250 top significant DEGs based on p-value < 0.o5 were considered to further analysis. Chang fold of all DEGs was less than 0.5 and more than 2. It was appeared that 60 DEGs were not characterized. 100 relevant genes were added to 190 characterized DEGs to construct interacted unit 286 nodes were recognized by STRING and the network including 44 isolated genes, one paired nodes and a main connected component (contains 240 nodes and 3893edes) was built (Figure 2). Degree distribution was fitted on y = ax^b^ equation where a and b were 11.865 and -0.459, respectively. Correlation and R-squared (computed on logarithmized values) were 0.870 and 0.458, respectively. As it is shown in the table 1, 5 hub nodes and 12 bottlenecks (top nodes based on betweenness centrality value) are identified. Surprisingly, all the bottlenecks are top nodes based on closeness centrality and only there are two hub-bottlenecks. Action network regarding 15 central nodes (characterized by top values of degree, betweenness, and closeness centralities) is shown in the figure 3. Expression action including up – regulation and down – regulation, activation and inhibition actions are illustrated in this map. 13 biochemical pathways related to the central nodes are determined; however, only 11 genes among 15 central individuals are involved (Table 2). Distribution of central nodes among biochemical pathways is tabulated in table 3.

## Discussion

Molecular mechanism of wide varieties of diseases is investigated via PPI network analysis and among large numbers of differential agents as like genes, proteins, and metabolites, few numbers with high impact on onset and development of diseases are identified. The introduced high impact biomolecules are suitable candidates for biomarker discovery (14, 15). In this study gene expression profiles of three grade III rectum cancer patients are compared with three ones in grade II. As it is shown, samples are matched statistically. 

**Table 3:** Distribution of central nodes among biochemical pathways

R	Gene name	Number of pathways
1	AKT1, EGFR,	11
2	EGF, TP53	9
3	CTNNB1	4
4	INS, SRC	3
5	RHOA	2
6	GAPDH, IL6	1

As it is shown in the figure 2 and table 1, network analysis revealed that 15 central nodes are involved in grade II into grade III transition in rectum cancer. Except AKT1 and PRDM10 the other nodes are hubs or central nodes based on closeness and betweenness centralities. Action map which is illustrated in the figure 3 showed that GMPS and DECR1, that are weak central nodes, have no regulatory effect on the other ones. PRDM10 the second hub-bottleneck has single activation edge on ACACA but the others interacted via compact activation, inhibition, and expression action with each other. As it is shown in table 2, 13 biochemical pathways in 5 groups are identified which are related to the central nodes. Group 3 includes only one pathway which is determines as colorectal cancer. AKT1, CTNNB1, RHOA, and TP53 are the four central genes (the genes whit high values of centrality parameters) that are involved in this pathway. For better understanding distribution of central nodes in pathways is shown in the table 3. Only 10 genes among 15 central nodes including AKT1, EGFR, EGF, TP53, CTNNB1, INS, SRC, RHOA, GAPDH, and IL6 are involved in the pathways. AKT1, EGFR, EGF, and TP53 have the most participation in the pathways. It seems that AKT1 plays crucial role in transition of grade II rectum cancer into grade II. Inhibition effect of AKT1 on TP53 and INS is highlighted in the figure 3. In many studies it is emphasized that loss of functional role of TP53 is a frequent event in cancer onset and development (16, 17). INS is an important metabolic hormone and is responsible for anabolic signals that promote tumor development. There is evidence that hyperinsulinemia is related to colon cancer. Bioactivity of insulin like growth factor-I increases by insulin. It is reported that this growth factor promotes CRC (18). However, inhibition of TP53 by AKT1 is corresponded with the oncogenic role of AKT1 but inhibition of insulin which plays a positive role in development of CRC by AKT1 is a paradox. To solve this paradox, we returned to the figure 3, AKT1 inhibits insulin while insulin activates AKT1. This relationship between AKT1 and insulin indicates that insulin promotes its oncogenic property via oncogene mediators such as AKT1. In a negative feedback AKT1 inhibits insulin. Considering this prominent role of insulin in rectum cancer, it can be concluded that the factors which increase insulin biosynthesis and secretion indirectly are involved in rectum cancer promotion. The main element in regulation of insulin level in body is glucose. Investigations indicate that glucose can elevate insulin level in blood by 4 fold. Beside this direct effect of glucose on insulin biosynthesis and secretion, it is found that high level of glucose leads to more stabilization of insulin mRNA which its stability is lower in the normal condition (19). This finding consists with the impact of life style on onset of rectum cancer. EGFR which transmits cell growth signals, plays significant role in cancer development. TGFα and EGF stimulate EGFR to induce cell growth signaling in normal condition and are involved in invasive and metastatic condition of cancers (20). Therefore, appearing of EGFR and its ligand EGF as crucial elements of rectum cancer is not a surprising finding. As it is shown in table 2, 6 numbers of central genes including AKT1, EGF, EGFR, GAPDH, IL6 and INS are involved in hypoxia inducible factor-1 signaling pathway. This combination of central genes relative to the enriched pathways includes maximum numbers of central genes. This pathway is responsible for delivery of oxygen and increment level of HIF-1 is correlated to tumor metastasis and angiogenesis. In response to hypoxia in tumors HIF-1 level increases (21). 

In conclusion it seems that AKT1, EGFR, and TP3 are suitable drug targets to prevent rectum cancer progression. The other point is high impact of life style especially diet regime which play crucial role in regulation of main players in the field of rectum cancer. 
